# Protective Role of Glutathione Peroxidase 4 in Laser-Induced Choroidal Neovascularization in Mice

**DOI:** 10.1371/journal.pone.0098864

**Published:** 2014-06-04

**Authors:** Murilo Felix Roggia, Hirotaka Imai, Tomoyasu Shiraya, Yasuo Noda, Takashi Ueta

**Affiliations:** 1 Department of Ophthalmology, Graduate School of Medicine and Faculty of Medicine, The University of Tokyo, Tokyo, Japan; 2 School of Pharmaceutical Sciences, Kitasato University, Tokyo, Japan; University of Florida, United States of America

## Abstract

**Purpose:**

To evaluate the influence of glutathione peroxidase 4 (GPx4) expression in retinal pigment epithelium (RPE)/choroid tissue using a mouse model of laser-induced choroidal neovascularization (CNV).

**Methods:**

In this study, GPx4^+/−^, GPx4^+/+^, and GPx4-overexpressing transgenic mice were created for comparison. The mRNA and protein expression of vascular endothelial growth factor (VEGF)-A in RPE/choroid tissue were evaluated before and after CNV induction by laser. Moreover, we investigated the changes in the VEGF-A mRNA level in RPE/choroid tissue in the CNV model that have not been clearly shown previously. Lipid peroxidation in RPE/choroid tissue was evaluated by immunohistochemistry using antibody against 4-hydroxy-2-nonenal. To investigate the protective role of GPx4, the size of laser-induced CNV was compared on day 7 among the mice expressing different levels of GPx4.

**Results:**

In the laser-induced CNV mouse model, laser treatment reduced the VEGF-A mRNA level in RPE/choroid tissue, while it increased the VEGF-A protein level. Evaluation of VEGF-A expression in RPE/choroid tissue of the GPx4^+/−^, GPx4^+/+^, and GPx4 transgenic mice revealed that GPx4 increased the VEGF-A protein level under physiological conditions (i.e., without laser treatment), while GPx4 suppressed the increase in the VEGF-A protein level under pathological conditions (i.e., after CNV induction by laser). In addition, GPx4 reduced the CNV size in a dose-dependent manner *in vivo.*

**Conclusions:**

GPx4 suppresses the increase in the VEGF-A protein level, which occurs during the development of pathological CNV, thus partly explaining the protective effect of GPx4 against CNV.

## Introduction

Age-related macular degeneration (AMD) is a prevalent disease worldwide, and it is considered a leading cause of irreversible visual loss [1]–[3]. Neovascular AMD is characterized by abnormal neovascular ingrowth from the choroidal vasculature toward the retinal pigment epithelium (RPE) and neurosensory retina, causing edema, bleeding, and disruption of photoreceptors. The involvement of vascular endothelial growth factor (VEGF) is well established in the pathogenesis of human neovascular AMD [4] as well as in the animal model of laser-induced choroidal neovascularization (CNV) [5].

On the basis of the results obtained using animal models, oxidative stress has also been implicated in the development of neovascular AMD [6]–[9]. On the basis of data showing that supplementation with antioxidant vitamins could suppress the development of neovascular AMD [10], oxidative stress is also considered to be involved in human neovascular AMD. Expression of antioxidant enzymes constitutes the key endogenous defense system against oxidative stress, and its implication in neovascular AMD has been reported following studies on superoxide dismutase 1-deficient mice [6], [8]. However, the possible involvement of other important antioxidant enzymes, including glutathione peroxidase 4 (GPx4), is not well understood.

GPx4, also known as phospholipid hydroperoxide glutathione peroxidase, is a member of the group of selenoproteins that have a selenocysteine amino acid residue at their enzymatic active site. GPx4 is ubiquitously expressed, and it directly reduces peroxidized phospholipids produced in cell membranes. In contrast to other GPx isoforms, GPx4 reduces complex lipid hydroperoxides, even when they are incorporated in biomembranes or lipoproteins [11]. GPx4 has been shown to be critically important because its ablation in mice leads to embryonic lethality at 7.5 days [12]. In addition, GPx4 ablation specifically in photoreceptors [13], cerebral neurons [14], vascular endothelium [15], or spermatocytes [16] causes severe pathological phenotypes.

In the present study, we aimed to evaluate the role of GPx4 in RPE/choroid tissue using a laser-induced CNV mouse model.

## Methods

All procedures were performed in accordance with the ARVO Statement for the Use of Animals in Ophthalmic and Vision Research and were approved by the Institutional Animal Research Committee of the University of Tokyo.

### Animals

Mice were maintained in a temperature-controlled room, wherein fresh water and rodent-specific diet were available *ad libitum.* We used GPx4^+/−^, GPx4^+/+^, and GPx4 transgenic mice on a C57BL/6 background. To obtain these mice expressing different levels of GPx4, first GPx4^+/+^ wild-type mice were bred with transgene-rescued GPx4 knockout mice GPx4^−/−^:Tg (GPx4) [16] to generate GPx4^+/−^:Tg (GPx4) and GPx4^+/−^ mice. Then, the GPx4^+/−^: Tg (GPx4) mice were bred with the GPx4^+/−^ mice to obtain GPx4^+/−^, GPx4^+/+^, and GPx4^+/+^: Tg (GPx4) mice for comparison.

### Laser-induced CNV model

CNV lesions were induced by laser photocoagulation as described previously [17]. A glass cover slip served as a contact lens. Diode laser (DC-3000; Nidek, Osaka, Japan) irradiation was delivered through a slit lamp (SL150; Topcon, Tokyo, Japan) to the mouse fundus between the major retinal vessels using a spot size of 200 µm, power of 200 mW, and exposure duration of 20 ms. Disruption of Bruch’s membrane was confirmed by central bubble formation immediately after photocoagulation. For each eye, 6 successful laser spots were created. After laser treatment, the mice were maintained on a physiological 12-h light cycle. On day 7, the mice were deeply anesthetized and perfused with fluorescein isothiocyanate dextran in PBS. Then, the mice were sacrificed, and the eyes were enucleated and fixed in 4% paraformaldehyde (PFA) for 20 min. RPE/choroid tissue was separated under a microscope and flat-mounted with the RPE facing up. The CNV area was measured on the basis of pictures analyzed by a blinded examiner using Photoshop CS3. CNV size was evaluated on day 7 because the size is considered to reach the maximum on approximately day 5–7 [5], [18]. In contrast, VEGF-A expression in RPE/choroid tissue was evaluated on day 3 (i.e., 72 h after laser treatment) when CNV is considered in the active stage of its development in order to evaluate the pathogenic role of VEGF-A.

### Real-time RT-PCR

RPE/choroid complexes were microsurgically isolated from the eyes. RNA from homogenized samples of RPE/choroid tissue was extracted using TRIzol reagent (Invitrogen, Carlsbad, CA), and cDNA was prepared using SuperScript III for RT-PCR (Invitrogen). Real-time PCR was performed using a Thermal Cycler Dice Real Time System (Takara Bio Inc., Shiga, Japan). Expression levels of each gene were normalized to those of GAPDH. The primer sequences used for real-time RT-PCR are listed in Table 1. We used 3 different primer pairs for VEGF-A and also a primer pair for VEGF 164 to determine the consistency of the results, because changes in the VEGF-A mRNA level in RPE/choroid tissue after CNV induction have not been well established in the literature [19], [20]. We tested 3 primer pairs used in previously published studies [19]–[21] and compared them to the primer pair that we designed. We first determined the validity of the primer sequences shown in the literature using Primer-BLAST and found errors in some of the published primer sequences. Therefore, we used primers with corrected sequences.

**Table 1:** Primers used in this study.

Primer pair	Sequences
Mouse VEGF-A primer pair 1	Forward: GTACCTCCACCATGCCAAGT
	Reverse: GCATTCACATCTGCTGTGCT
Mouse VEGF-A primer pair 2 [20]	Forward: AGGCTGCACCCACGACAGAA
	Reverse: CTTTGGTCTGCATTCACATC
Mouse VEGF-A primer pair 3 [21]	Forward: AGCCGAGCTCATGGACGGGT
	Reverse: AGTAGCTTCGCTGGTAGACATC
Mouse VEGF 164 [19]	Forward: GCCAGCACATAGGAGAGATGAGC
	Reverse: CAAGGCTCACAGTGATTTTCTGG
Mouse GAPDH	Forward: CACATTGGGGGTAGGAACAC
	Reverse: AACTTTGGCATTGTGGAAGG

### Protein expression analysis

The isolated RPE/choroid complexes were placed in 100 µL RIPA buffer and homogenized at 4°C. Protein concentrations were determined using a Coomassie (Bradford) Protein Assay Kit (Thermo Scientific, Waltham, MA). For western blot analysis, total protein extracts from RPE/choroid samples were separated on SDS-PAGE gels and transferred onto nitrocellulose membranes followed by blocking with 5% non-fat dry milk in TBS-T (Tris-buffered saline with 0.1% Tween-20). Incubation with primary antibodies was performed for 6 h in TBS-T containing 5% non-fat dry milk. The primary antibodies used were mouse or rabbit antibodies against β-actin (Sigma, St Louis, MO) and against GPx4 obtained from a primary hybridoma [22]. The membranes were incubated with anti-mouse/rabbit horseradish peroxidase-labeled secondary antibody (Amersham Biosciences, Chalfont St Giles, UK) for 1 h. The washed membranes were further developed with ECL Plus Western Blotting Detection Reagents (GE Healthcare, Piscataway, NJ). The protein level was calculated by normalization to the β-actin level.

ELISA was used for the evaluation of VEGF-A protein, according to the manufacturer’s instructions (R&D Systems, Minneapolis, MN).

### Immunohistochemistry

Enucleated eyeballs were fixed in 4% PFA for 6 h, embedded in paraffin, and the posterior retina was cut into 5-µm-thick sections. Slides were first incubated with blocking solution (2% normal goat serum) overnight, and then with primary antibodies at room temperature for 3 h and secondary antibodies for 1 h. The sections were then coverslipped with mounting medium. For immunostaining, the primary antibodies used were mouse monoclonal antibodies specific to 4-hydroxy-2-nonenal (4-HNE) (JaICA, Shizuoka, Japan). Hematoxylin-eosin staining was used to reveal the morphology of the retina and RPE/choroid tissue. The intensity of immunofluorescence in RPE/choroid was evaluated using Image-J software.

### Statistics

All statistical analyses were performed using JMP10 software (SAS Institute Inc., Cary, NC). For comparison between 2 unpaired groups, Student’s t-test was used. For comparison among 3 or more groups, one-way analysis of variance (ANOVA) was performed, followed by Tukey’s test. The level of significance was set at *P*<0.05.

## Results

### Change in VEGF-A expression in RPE/choroid tissue after CNV induction

**Figure 1:**
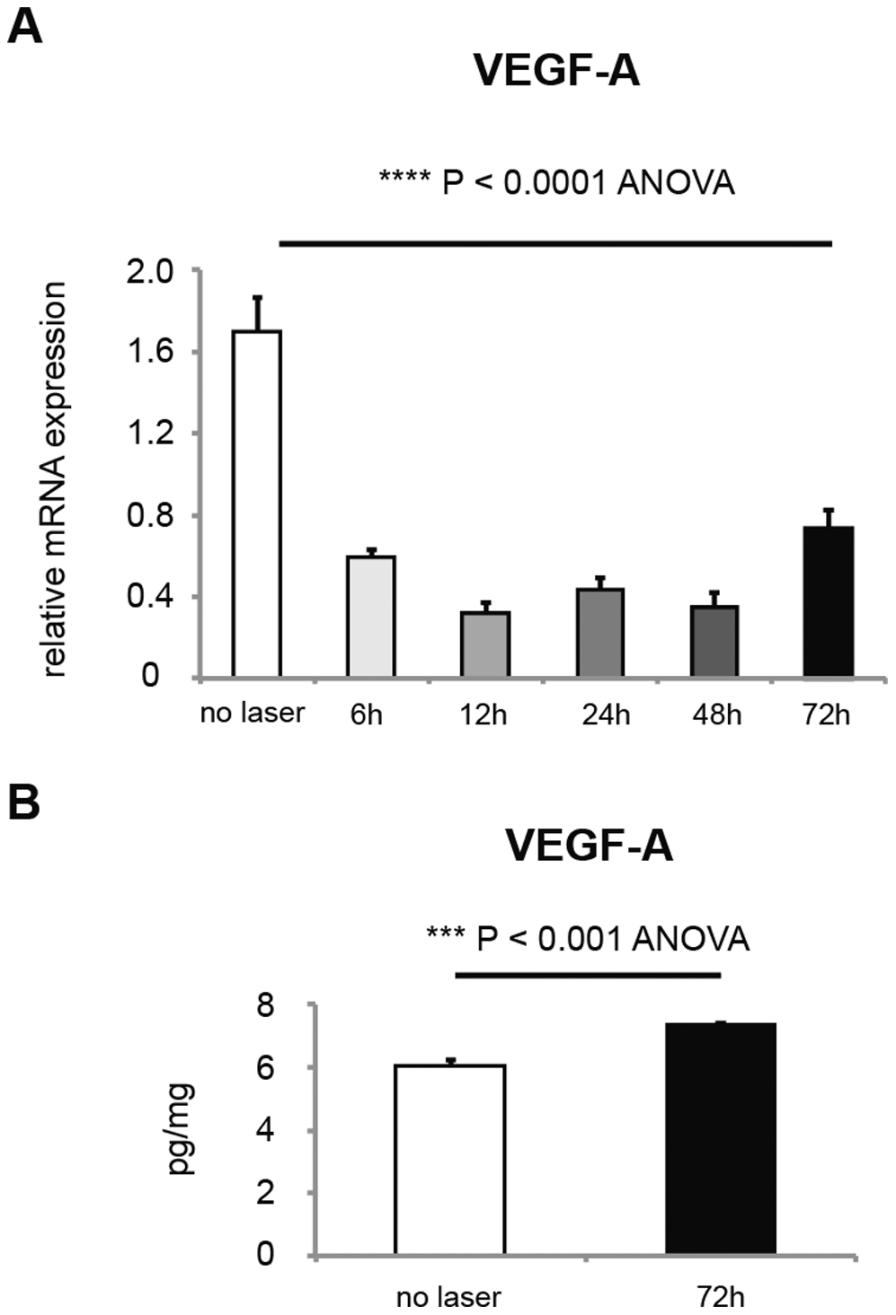
VEGF-A expression in RPE/choroid with laser-induced CNV. **(A)** Relative VEGF-A mRNA level in RPE/choroid of wild-type mice before and after induction of CNV model at 6, 12, 24, 48 and 72 hours (mean±SEM, n = 10–21, **** P<0.0001). (**B**) VEGF-A protein expression in RPE/choroid of wild-type mice before and after 72 h of CNV induction by laser, measured by ELISA (mean±SEM, n = 4, *** P<0.001).

In the laser-induced CNV model, there have been contradictory reports regarding the VEGF-A mRNA level in RPE/choroid tissue, while there is an established consensus concerning the increased protein expression [19], [20]. Therefore, in the present study, we first evaluated mRNA (Figure 1A) and protein (Figure 1B) expression in RPE/choroid tissue of mice with laser-induced CNV. Unexpectedly, we found that the VEGF-A mRNA level in RPE/choroid tissue was reduced by CNV induction throughout the first 72 h during CNV development. However, consistent with CNV development, the VEGF-A protein level was higher at 72 h after laser treatment. The unexpected downregulation of VEGF-A mRNA was also confirmed by performing RT-PCR using other primer pairs that were used in previous studies (Figure S1) [19]–[21]. These results might indicate a complex mechanism in the regulation of VEGF expression in RPE/choroid tissue.

### Generation of GPx4^+/−^, GPx4^+/+^, and GPx4-overexpressing mice

**Figure 2:**
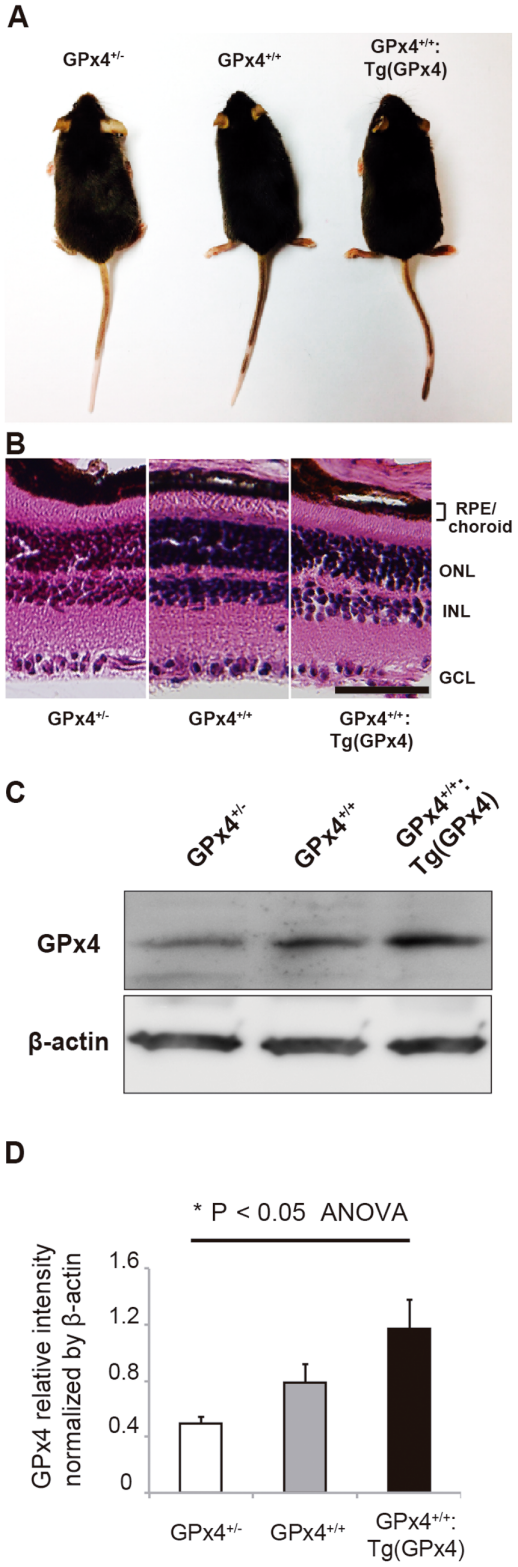
GPx4 expression in RPE/choroid. (**A**) Similar appearance of GPx4^+/−^, GPx4^+/+^ and GPx4^+/+^:Tg (GPx4) mice at 3 months of age expressing different levels of GPx4. (**B**) Hematoxylin-eosin staining of the retina and RPE/choroid of the mice expressing different levels of GPx4. (ONL; outer nuclear layer, INL; inner nuclear layer, GCL; ganglionar cells layer, Scale bar; 30 µm). (**C**) Western blot analysis of β-actin and GPx4 protein expression in the RPE/choroid. (**D**) Statistical evaluation for the comparative difference in GPx4 protein in RPE/choroid (mean±SEM, n = 5)

**Figure 3:**
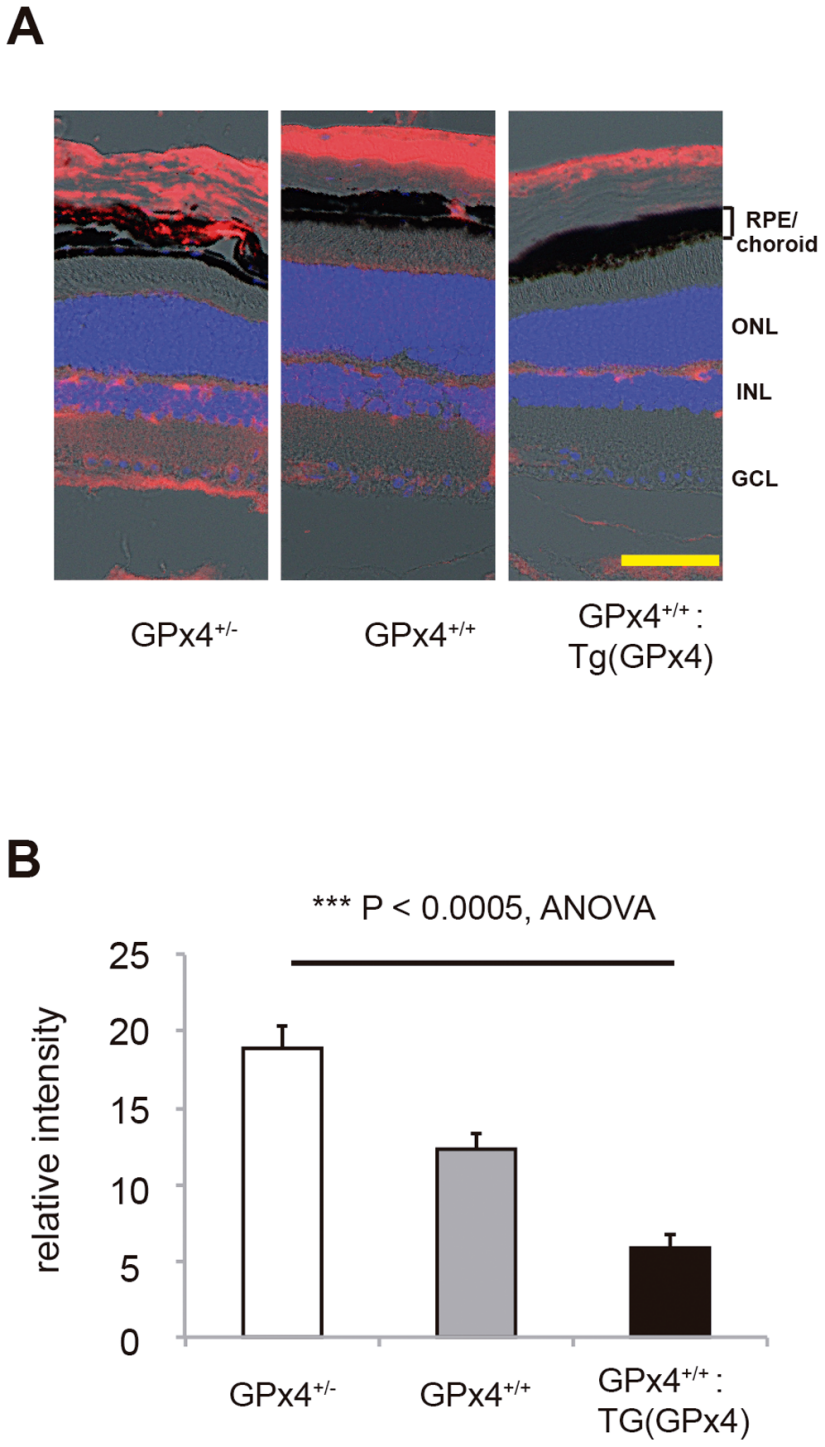
Accumulation of peroxidized lipid. (**A**) Immunoreactivity to 4-hydroxi-2-neonenal (4-HNE, red) in the retina and RPE/choroid of mice expressing different levels of GPx4. Nuclei was counterstained with 4,6-diamidino-2-phenylindole (DAPI, blue). Scale bar, 30 µm. (**B**) Statistical evaluation of the immunofluorescence for 4-HNE in RPE/choroid (mean±SEM, n = 4).

Figure 2 shows the generation of 3 types of mice expressing different levels of GPx4. All these mice grow normally (Figure 2A) and display normal morphology of the retina and RPE/choroid tissue in all three genotypes (Figure 2B). However, western blot analysis revealed different expression levels of GPx4 protein in RPE/choroid tissue among the 3 types of mice (Figure 2C), which confirmed the validity of using these mice to test the role of GPx4 in RPE/choroid tissue. We also confirmed that GPx1 and GPx2 protein expression in RPE/choroid is similar in these mice (Figure S2). Using antibody against 4-HNE revealed that the accumulation of oxidized lipid in RPE/choroid tissue was most abundant in the GPx4^+/−^ mice and least in the GPx4-overexpressing [i.e., GPx4^+/+^:Tg (GPx4)] mice (Figure 3).

### Laser-induced CNV model

**Figure 4:**
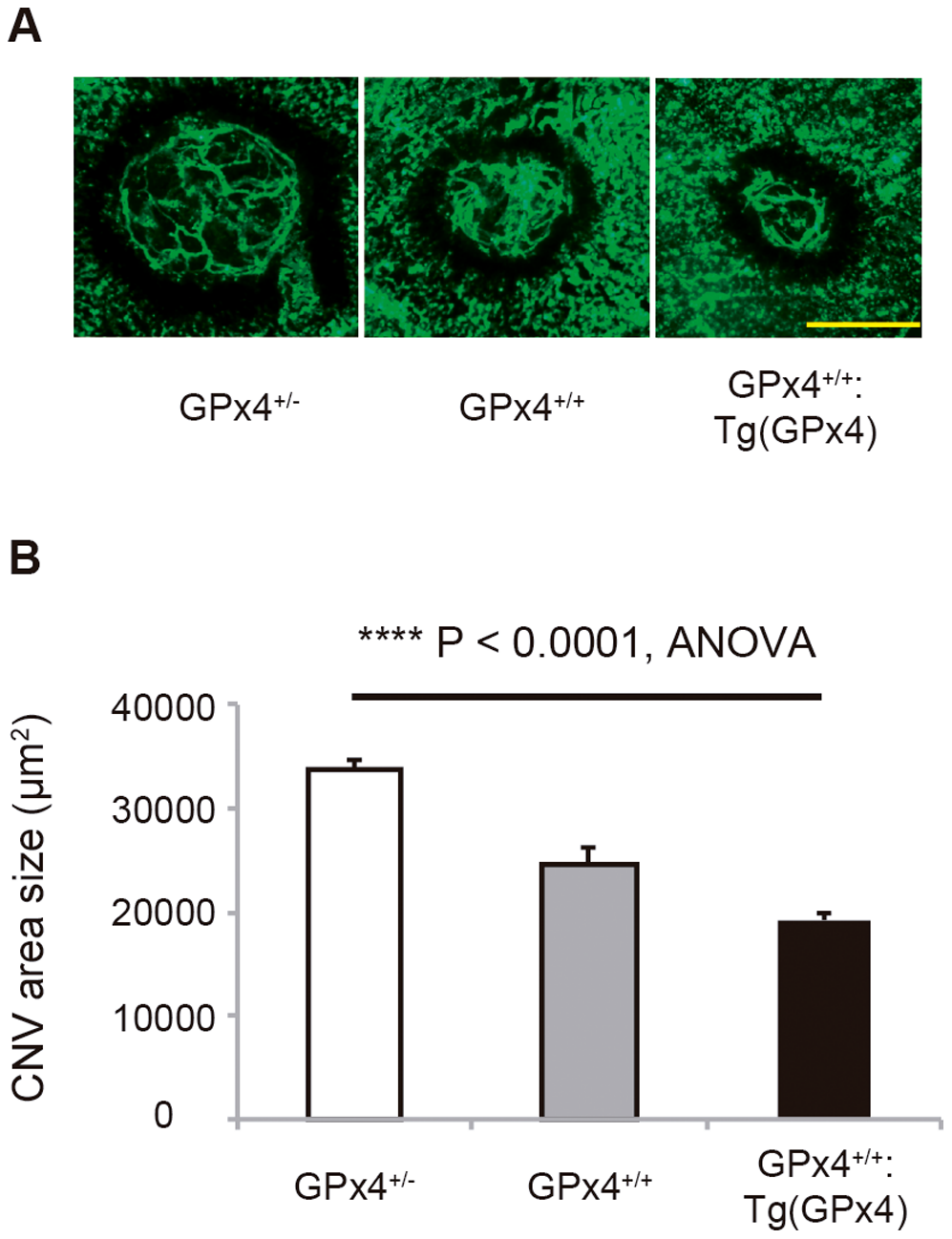
Laser-induced CNV model. CNV area size was evaluated on flatmount on day 7. (**A**) CNV induced by laser in the mice expressing different levels of GPx4. Scale bar, 500 µm. (**B**) The area size of the CNV in these 3 types of mice (µm^2^, mean±SEM, n = 4–5, **** P<0.0001).

**Figure 5:**
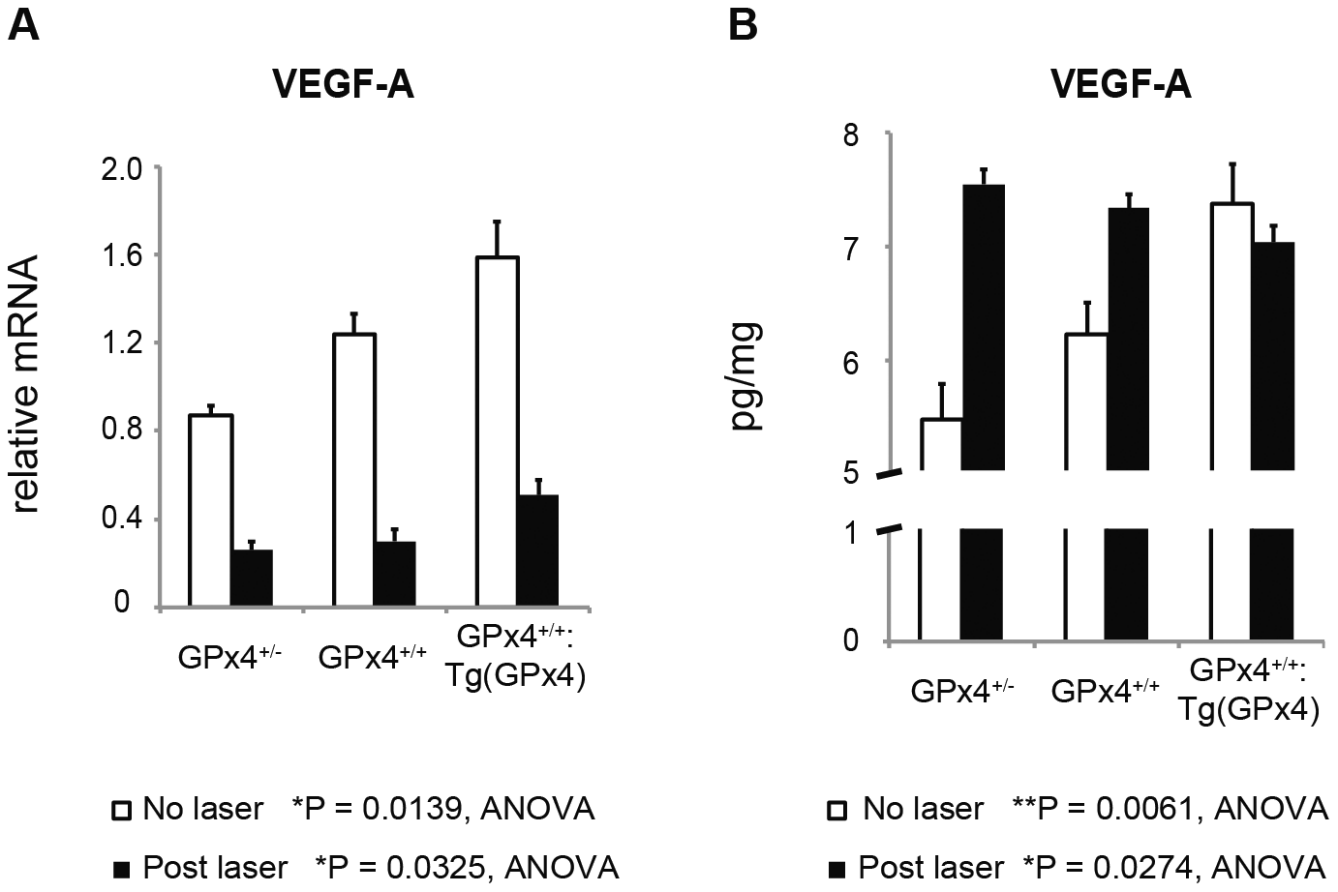
VEGF-A expression in RPE/choroid before and after CNV induction. (**A**) Relative VEGF-A mRNA level was measured by real time RT-PCR before and 3 days after the CNV induction by laser (mean±SEM, n = 4–6). (**B**) VEGF-A protein expression in RPE/choroid was measured by ELISA before and 3 days after the CNV induction (mean±SEM, n = 4–6).

Next, we evaluated the influence of different levels of GPx4 on CNV development using a laser-induced CNV mouse model (Figure 4). With increasing GPx4 expression, CNV size was reduced significantly on day 7 when the size is considered to reach its maximum [5], [18]. We also investigated VEGF-A expression on day 3 when CNV is considered in the active stage of its development. As confirmed in the wild-type mice, VEGF-A mRNA was downregulated in all 3 groups. In contrast, with or without laser treatment, the VEGF-A mRNA level in RPE/choroid tissue was significantly higher in mice expressing higher levels of GPx4 (Figure 5A). However, VEGF-A protein expression exhibited a more peculiar pattern (Figure 5B). In RPE/choroid tissue without CNV induction, the VEGF-A protein level was higher in mice expressing more GPx4 (*P* = 0.0061 by ANOVA). Three days after CNV induction, the VEGF-A protein level was upregulated more in mice expressing lower levels of GPx4. As a result, the VEGF-A protein level was significantly higher in mice expressing less GPx4 than in mice overexpressing GPx4 (*P* = 0.0274 by ANOVA). In the GPx4^+/−^ and GPx4^+/+^ mice, the difference in the VEGF-A protein level between mice with and without laser-induced CNV was significant (*P* = 0.0001 and *P* = 0.0022 by Student’s *t*-test, respectively). In mice overexpressing GPx4, the VEGF-A protein level did not differ significantly between mice with and without laser-induced CNV (*P* = 0.45).

## Discussion

The results of the present study showed that GPx4 influences VEGF-A expression in RPE/choroid tissue under both physiological and pathological conditions, and that it confers protection against CNV development. In the present study, we investigated the role of GPx4 on the basis of VEGF-A because the importance of VEGF-A has been well established through studies both in animals [5] and in patients with CNV [4]. However, the relationship between antioxidant enzymes, VEGF-A, and CNV has not been clarified to date.

We used GPx4^+/−^ mice and GPx4-overexpressing mice to evaluate the importance of GPx4. This is because we were unable to produce live conditional knockout mice in which GPx4 expression was abrogated specifically in RPE. This could have been because we used RPE65-Cre mice where Cre was expressed from the early stage of development. However, by using mice expressing different levels of GPx4, we were able to clearly demonstrate the protective role of GPx4 against CNV.

First, we analyzed the time course of VEGF-A mRNA expression in RPE/choroid tissue. Although increased VEGF-A protein expression in mouse RPE/choroid tissue after CNV induction has been well established, the change in the VEGF-A mRNA level has not been thoroughly investigated. We found only a few reports describing the VEGF-A mRNA level in mice with laser-induced CNV. In two reports, the VEGF-A mRNA level increased 3 days after CNV induction [19], [23] while in another report, no change was apparent during 1 week after CNV induction [20]. In a recent comprehensive review on the methodology of CNV model, the change in VEGF-A mRNA was not discussed [5]. In the present study, using several different primer pairs, we confirmed that VEGF-A mRNA is downregulated after CNV induction by laser. Because RPE/choroid tissue consists of heterogenous cellular components including RPE and choriocappilaris, it is difficult to discuss the change in mRNA and protein of VEGF-A in specific cellular populations. However, the discrepancy in the level of mRNA and protein may warrant the evaluation of post-translational regulation of VEGF.

In line with the difference in GPx4 expression, we found a dose-dependent increase in VEGF-A protein expression in RPE/choroid tissue under physiological conditions. In contrast, as GPx4 expression increased, the increase in VEGF-A protein expression mediated by laser-induced CNV was suppressed. As a result, after CNV induction, the VEGF-A protein level was reduced in the GPx4-overexpressing mice. Under physiological conditions, the decreased and increased VEGF-A protein expression in the GPx4^+/−^ and GPx4-overexpressing mice, respectively, were unexpected results. However, these changes could be associated with the established importance of VEGF that is known to maintain the physiological choroidal vasculature [24]. Furthermore, VEGF overexpression itself did not induce pathological CNV [25]. On the other hand, the situation was drastically different in RPE/choroid tissue after CNV induction. The VEGF-A protein level was significantly upregulated by CNV induction in the GPx4^+/−^ mice, while the level did not significantly change in the GPx4 transgenic mice. This result is consistent with the expectation that GPx4 could confer protection against CNV growth.

In the present study, we evaluated the importance of GPx4 in RPE/choroid tissue using a laser-induced CNV model. In previous studies on superoxide dismutase 1 (SOD1), SOD1 knockout mice reportedly developed numerous age-related changes in RPE/choroid tissue including naturally occurring CNV [6], [21] or ischemic retinopathy [8]. Protective role of GPx4 against the oxidative stress in the retina was confirmed [26]. However, the effect of an antioxidant enzyme on laser-induced CNV or of VEGF-A expression in RPE/choroid tissue is not well understood. In a study evaluating the role of thioredoxin 1 [27], laser was applied at the threshold intensity and the ratio of the number of CNV to the number of laser spots was observed. Because evaluating CNV size is the most widely accepted methodology for the laser-induced CNV model [5], [18], the present study, to our knowledge, is the first to demonstrate the importance of an endogenous antioxidant enzyme in a laser-induced CNV model with VEGF-A expression in RPE/choroid tissue.

In summary, we demonstrated that GPx4 influences VEGF-A expression in RPE/choroid tissue under both physiological and pathological conditions and confers protection against CNV development *in vivo*. Based on the results of the present study, we propose that GPx4 could be a potential target for CNV treatment.

## Supporting Information

Figure S1 Change in VEGF-A mRNA level in RPE/choroid after CNV induction in wild-type mice. For the same samples shown in Figure 1, different primer pairs for VEGF-A was used for real-time RT-PCR. Sequences of the primers are shown in Table 1. **(A)** A primer pair 2 [20] was used. **(B)** A primer pair for VEGF 164 [19] was used. **(C)** A primer pair 3 [21] was used. (mean ± SEM, n  =  10–21 per group).
(TIF)

Figure S2 Protein expression of GPx1 and GPx2 in the RPE/choroid. **(A)** Western blot analysis of β-actin and GPx1/2 protein expression in the RPE/choroid. **(B)** Statistical evaluation for the comparative difference in GPx1/2 protein in RPE/choroid (mean ± SEM, n  =  5).
(TIF)
