# Supplementary figures and images for: Protective Role of Glutathione Peroxidase 4 in Laser-Induced Choroidal Neovascularization in Mice
Source: PLoS One. 2014 Jun 4;9(6):e98864. doi: 10.1371/journal.pone.0098864 (PMC4045803; doi:10.1371/journal.pone.0098864)

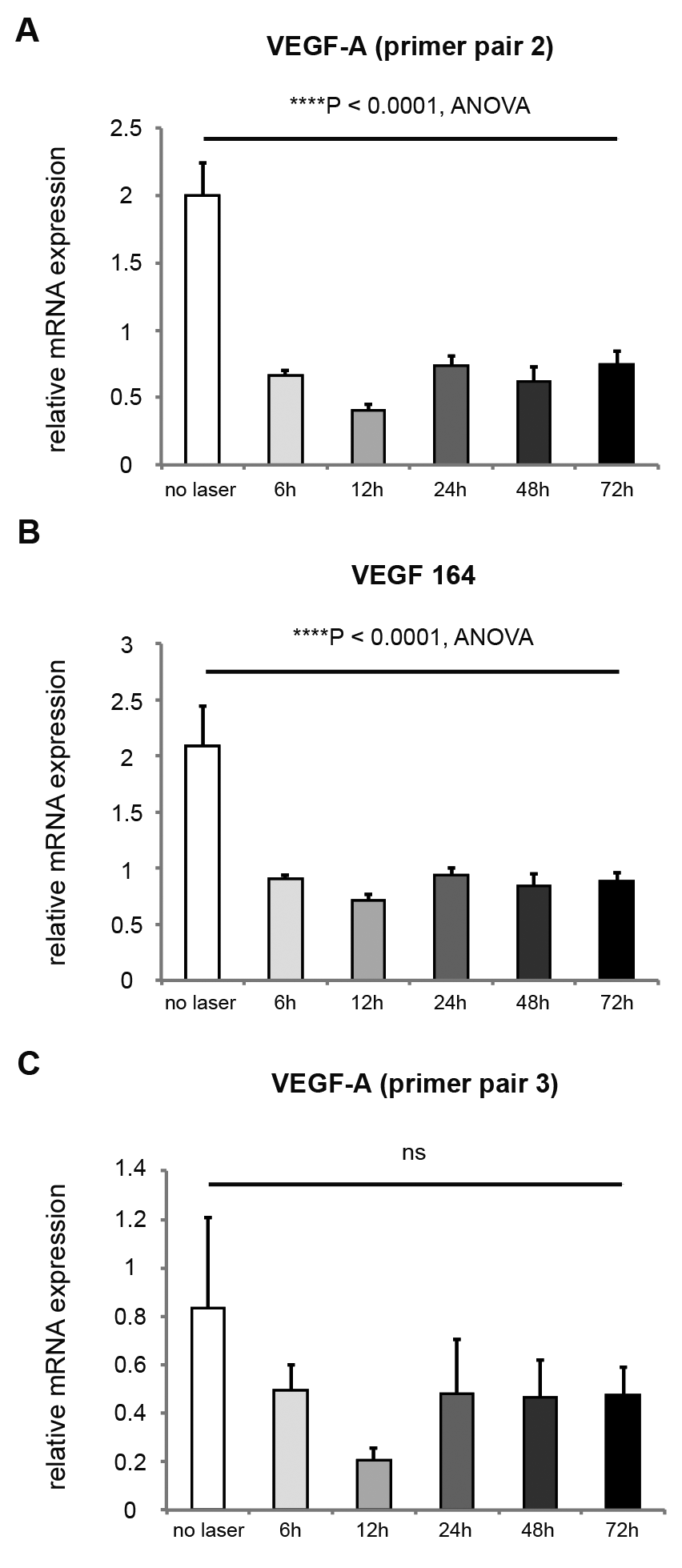

Supplement: Figure S1 — Change in VEGF-A mRNA level in RPE/choroid after CNV induction in wild-type mice. For the same samples shown in Figure 1, different primer pairs for VEGF-A was used for real-time RT-PCR. Sequences of the primers are shown in Table 1. (A) A primer pair 2 [20] was used. (B) A primer pair for VEGF 164 [19] was used. (C) A primer pair 3 [21] was used. (mean ± SEM, n = 10–21 per group). (TIF) [file pone.0098864.s001.tif]

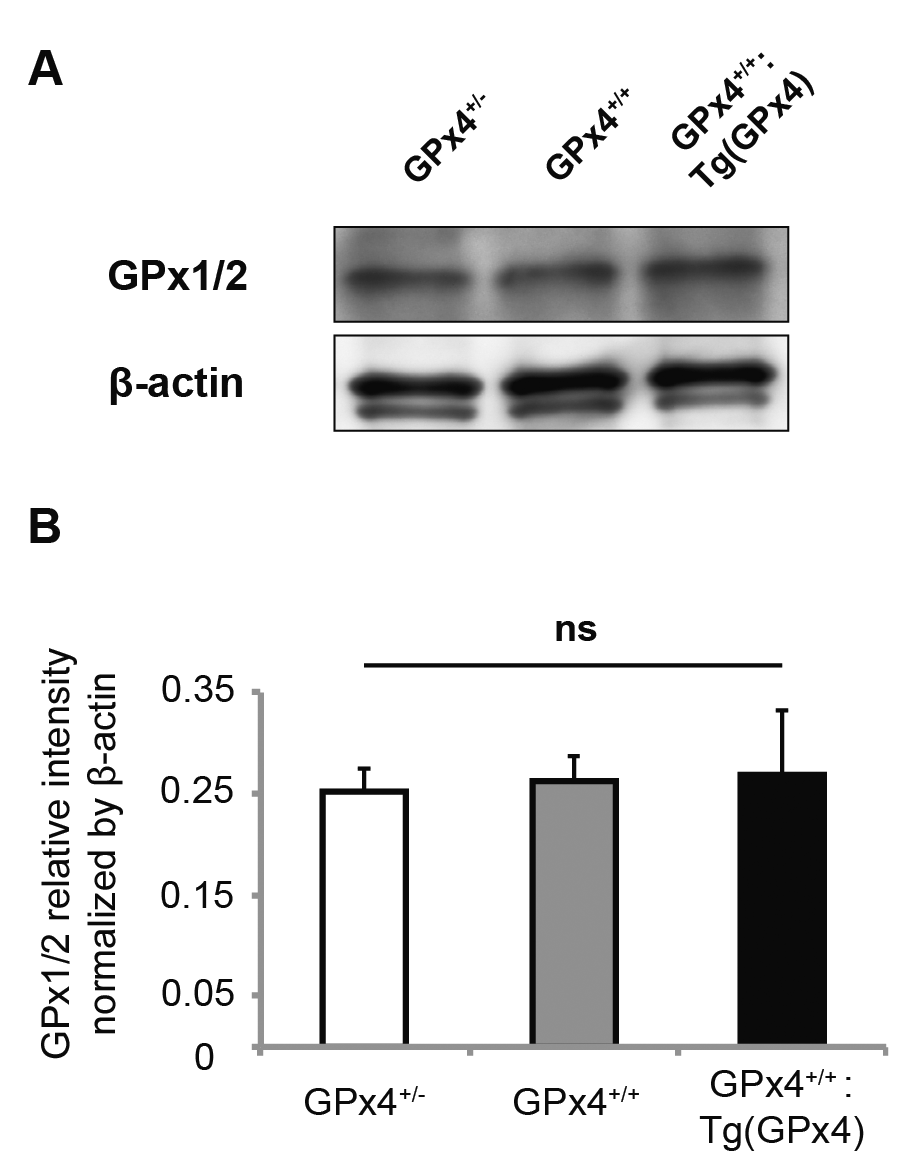

Supplement: Figure S2 — Protein expression of GPx1 and GPx2 in the RPE/choroid. (A) Western blot analysis of β-actin and GPx1/2 protein expression in the RPE/choroid. (B) Statistical evaluation for the comparative difference in GPx1/2 protein in RPE/choroid (mean ± SEM, n = 5). (TIF) [file pone.0098864.s002.tif]
